# Mr. Macilwain on Porrigo

**Published:** 1834-04-01

**Authors:** 


**CLINICAL OBSERVATIONS ON THE CONSTITUTIONAL ORIGIN OF THE VARIOUS FORMS OF PORRIGO; COMMONLY KNOWN BY THE NAMES OF SCALD-HEAD, TINEA, RING-WORM, &c.: WITH DIRECTIONS FOR THE MORE SCIENTIFIC AND SUCCESSFUL MANAGEMENT OF THIS USUALLY OBSTINATE CLASS OF DISEASES, BY A TREATMENT CONSISTING OF AN APPROPRIATE MODIFICATION OF THOSE PRINCIPLES FIRST PARTICULARLY PROMULGATED BY MR. ABERNETHY. By *George Macilwain*, Surgeon to the Finsbury Dispensary, &c. Octavo, pp. 83.**

THE uncertain and highly unsatisfactory results which have heretofore attended the management of cutaneous diseases in general, though conducted with the most scrupulous adherence to the dominant pathology of the day, in relation to this special department of the practice of medicine, have impressed upon the profession at large the urgent necessity which exists for a narrow and rigid examination of the validity of those principles and views, which have directed the treatment of a class of affections composing so large a proportion of those commonly met with in private, and more particularly in dispensary practice.

The author of the little volume which forms the subject of the present notice is already not unfavourably known to his professional brethren, in connexion with other productions of his pen, which have been briefly reviewed in this Journal.* Before proceeding to submit to the reader an analysis of this last, but not least important of Mr. Macilwain's publications, we deem it advisable to advert to the appointment which he holds, as Surgeon to the Finsbury Dispensary, in which capacity ample opportunities, we conceive, must have been afforded him for submitting to the touchstone-test of experience the comparative value and success attendant on the various modes of treatment recommended in the works of British and Continental authors, who have treated systematically of diseases of the skin.

The Finsbury Dispensary, "as large as any, if not the largest in the Metropolis" (p. 21), over which our author presides, relieves about five thousand patients annually. These, in general, belong to the lowest classes of the dirty and ill-fed poor, and may be supposed to be by no means very favourable subjects for promoting the success of any remedial measures. It would nevertheless appear, that the modified constitutional plan of treatment

---

* Vide Medico-Chirurgical Review for July, 1827.

has, in the practice of Mr. Macilwain, proved eminently successful in curing a tribe of skin diseases, hitherto, in the experience of many practitioners, regarded as perhaps the most obstinate and unmanageable of any ; we allude to scald-head, tinea, ring-worm of the scalp, or by whatever other epithet the various forms of porrigo may be styled. We are confident that the views and treatment entertained and recommended in Mr. Macilwain's little volume will receive, as they merit, the attention of many classes of the profession.

In a temperately-written preface, our author advocates the importance of those views promulgated by the late Mr. Abernethy—" on the Constitutional Origin of Local Diseases." He thinks, and many of the present day may no doubt concur with him, that these views have not yet received an application commensurate with their claims. Many modifications of Mr. Abernethy's principles, as applied to the relief of individual diseases, probably yet remain to be discovered. The reader is next apprized by our author, that he has good reasons for entertaining the assurance, that the great surgeon was not aware of the example now afforded, of the influence and value of his views when applied to the treatment of porrigo in its various forms ; and which, " in the absence of yet less matured illustrations," he at present submits to the profession. But we must hasten, without farther preamble, and present a summary of the contents of the treatise under consideration.

Skin diseases, being generally more or less complicated with some kind of constitutional disturbance, *à priori* we might be led to anticipate that, in proportion as the pre-existing or consequential disturbance was great, or comparatively trifling—the local disease in the one case would be less, just as, in the other, it would be more, amenable to the influence of remedial measures. Mr. Macilwain's experience has informed him that the state of the fact is widely different, and that a result apparently so contrary to all rational anticipation appears to admit of the following explanation :—

In some diseases the formidable nature of the general disturbance not only compels us to direct our attention to it, but often with an anxiety which renders us absolutely regardless of circumstances of minor import ; as happens in acute forms of the Exanthemata.—Other diseases are too manifestly accompanied by a bad state of health to allow of their connexion with it to be overlooked or mistaken ; a third set are so obstinate, so pertinaciously annoying, and are attended with so little local alteration, that this disproportion alone suggests the possible influence of a cause not to be discovered in the seat of its local manifestation. These circumstances (and as it appears to me, very much in the way here represented) have led physicians and surgeons already to treat the majority of cutaneous diseases by means directed to the improvement of the general health.*

There are however still, cutaneous affections in which the disturbance is neither formidable, alarming, nor very plainly developed, and in which its very existence, because often unattended by any very obvious indication, is overlooked and merged in the contemplation of the more prominently troublesome and disgusting peculiarities of their local characters, and wherein the treatment has been confined to the removal of these, regardless of the constitutional disturbance

* " The consideration of the whole of the diseases of the skin, strongly supports this view of the subject ; as examples, I may refer to
The Exanthemata, Lichen, Strophulus, Prurigo, which are in the order which I have placed their illustrations of these observations."

on which they depend. This is strongly exemplified in the treatment usually pursued in the various forms of Porrigo, and the result is just what sounder views of disease must lead us to expect; viz.—that with the exception of specific malignant diseases, they are the most obstinate of all cutaneous affections.

This appears to me to be a brief but true account of the state of the practice with regard to Porrigo; and I can only say, that if it be in any sense an overdrawn picture, it has not been occasioned by supineness on my part, in endeavouring to ascertain the real state of the case." 8.

It would be premature on our part either to assent or disagree with the tenour or bearing of these remarks, until we see their application, as enforced in connexion with the modified plan of treatment recommended by Mr. Macilwain, who confidently assures his readers, that if afforded a fair trial, the result will be such as not to create disappointment.

In the second chapter, we find some observations of a less general nature, on the "local characters and constitutional origin of porrigo," the various forms of which, known by the names of scald-head, tinea capitis, ring-worm, &c. may, from their local characters, be considered as "inflammations of certain portions of the skin, terminating in the formation of pustules."

Willan and Bateman, who, as most no doubt are aware, were guided in their classifications of diseases of the skin by the external or local characters, presented by this class of affections, very properly referred the different species of porrigo to the order "pustulæ," in their systematic arrangements. Some more recent authors have objected to porrigo *decalvans* being classed with the genus porrigo, from its never being attended with the formation of pustules. Mr. Macilwain, at pages 10, 25, and 26, ranges himself with those who have joined issue against the learned Bateman's questionable judgment on this point. Our author nevertheless, while urging the impropriety of such an alliance, for the reason just mentioned, does not regard the arrangement as disadvantageous or objectionable, when the state of the constitution is attended to, by which diseases of this kind are generally accompanied.

In a practical point of view, since nosologic distinctions in any class of diseases are often very unimportant, and, with reference to those designated by the common term "porrigo," mere nominal distinctions may be disregarded altogether; forasmuch as, all of them are easily relieved by treatment conducted on a common principle; though of course differing in its details. The grand principles here referred to are—regulating the digestive organs by a particular diet; and by the use of medicines, directed to ensure a healthy condition of the various secretions.

To revert, however, to the infinite diversity of forms and appearances which porrigo may assume, the practitioner need scarcely be reminded that skin diseases, no more than others, must be expected to tally to a nicety, with delineations in highly finished plates. Thus, affections forming the subject of Mr. Macilwain's treatise are usually presented in dispensary practice, or in the dwellings of the poor, "obscured by vast quantities of matted hair mixed with considerable incrustation, the offensive accumulation of several days, weeks, or even in some cases, months. The removal of all this discovers a red shining portion of inflamed skin, on which are seen numerous groups of small points which are to be considered as new pustules. When the disease is in progress of cure, these points are no longer observable. If, on the contrary, the disease is to continue, the pustules enlarge, become more or less confluent, and form incrustations which, on being thrown off or removed, again discover other pustules proceeding in the same manner." Ample details of the successive appearances of these common affections are given in the works of Willan, Bateman and Alibert.

We come however to that part of Mr. Macilwain's work, where he states, that so far as he has been able to learn, these affections have by others been treated almost entirely by local applications. He does not deny that aperients may have "occasionally" been administered, nor that dietetic cautions have been offered; but he contends, so far as his observations have gone, "no systematic attempt to regulate the diet has hitherto attended the practice." Cleanliness and local applications, though not unimportant, are yet merely subsidiary, in cases of obstinacy. Dr. Bateman, as is pretty well known, recommended tonics and particular diet in "particular cases;" yet, he is not regarded as worthy of being named an exception to the truth of the remark made. We may be allowed however to observe, that M. Alibert, so far back as 1798, or early in the current century, in the letter-press accompanying his highly finished delineations of forms of tinea, witnessed by him in St. Louis' Hospital, dwells at considerable length on the great want of success, from any treatment exclusively local; and prides himself, (as he rarely fails to do, when an opportunity is afforded,) in first seeing and urging the necessity of attending to age, temperament, season of the year, diet, together with close attention to the general health of patients, &c. This reference is offered only by the way, for we by no means disagree with our author, when he asserts that no writer before him had inculcated diet as among the chief, if not the chief point, to be systematically attended to, in treating porrigo.

Prosecuting our perusal and analysis, we find M. Alibert's opinions and experience, quite opposed by that of Mr. Macilwain, as to the contagious nature of porrigo. Most of our readers may be aware of the *outré* opinions and the still stranger practice broached and resorted to by Alibert and others of the French school, from regarding tinea and skin diseases generally in the light rather of conservative efforts and salutary crises of Nature, than diseases properly so called. When such was M. Alibert's creed, we need not express any surprise that in accordance with *his* philosophic views, he should have sought to profit by the interpreted hint thus afforded; and endeavoured, in many diseases, to excite artificially some skin affection, so as to *localise* the effects of constitutional disturbance; the tendency of which, if allowed to run its injurious course, must, *ex necessitate,* prove infinitely more injurious than a local disease. We actually find that children labouring under enteritic affections were *inoculated* with the mucus and purulent matter of scald head! This monomania for inoculation was indulged upon a liberal scale; yet the results of the operation, though practised with anxious attention, failed again and again, nay, oftener failed than succeeded in producing a skin disease in the punctured locality, as looked, and devoutly wished, for, by M. Alibert! Now, it was this general want of success in artificially exciting tinea, that led the "skin" physician of the Hospital of St. Louis to consider as very questionable, if not utterly to be set aside as untenable, the opinions of the contagious or infectious nature of the disease. We have considered it a duty to advert, however cursorily, to the opinions of one of the great French authorities on this question, which is any thing surely but of minor importance, when we bear in mind how much the business of education and the interests of boarding-school teachers are interfered with the instant a well-marked case of porrigo appears among the pupils. Much conflicting opinion has been originated, we have good reason to know, owing to ring-worm or tinea capitis being held by one medical party, consulted for the interests of the teacher, as not communicable by contact or social intercourse; while again the opposite opinion has been given on behalf of the parents and children by their medical referee. The profession will again and again be consulted on such occasions as they have already been. Looking not at exceptions, but at the broad general facts relating to the extension of porriginous diseases among the members of private families and boarding-houses, we do not think the slightest doubts can be entertained of such diseases being generally, if not uniformly, communicable by contact with those already affected. We leave this somewhat disputed topic with a pertinent extract of special and general import from the work before us, the bearings of which are sufficiently comprehensive.

"If there be any one thing clear about the often-discussed subject of infection and contagion, it is, that there is a certain condition of the system favourable to the acceptance of the contagion. On no other grounds than this admission can the various phenomena connected with this extensive subject admit of explanation; and it is no argument in support of a denial of this condition, to say that we are ignorant of any mode by which it can be discovered. Its existence is proved by the consideration of the facts presented both by those who receive, and by those who resist contagion. Indeed, were we to conclude that diseases were local because they occurred in individuals exposed to contagion, who were otherwise *apparently* healthy, I conceive that we could scarcely adopt an hypothesis more at variance with facts almost daily presented to us, or leading to more untenable or mischievous conclusions." 17.

From the extract just given, it will likewise be seen, that Mr. Macilwain disagrees with Dr. Bateman's remark, of porrigo being sometimes a purely local disease. The solitary fact of children, otherwise apparently in the enjoyment of good health, receiving the disease by contagion, assuredly is a premise, not sufficiently satisfactory, from which to deduce, as Bateman has done, such a summary conclusion.

Constitutional disturbance is not always equally palpable to the senses of experienced practitioners; and local diseases which ordinarily afford but obscure indications of the sources whence they spring, at other times most "glaringly expose to us, a fact in their character, which, under less formidable circumstances, had escaped our penetration" *(p. 15)*. This is a fact, which the experience of every-day practice must have inculcated on the attention of most of our profession. Diseases ushered in by obscure or inappreciable indications of general disorder often, subsequently, have their connexion with constitutional disturbance sufficiently attested; and no sooner has local irritation, or disease, been developed, than the previous general tumult has subsided. Knowledge of this induced the old practitioners, of the observant class, to dread "repulsion of the humours," as their pathology led them to express themselves. In all cases of *ulcered* legs, every day presented at our public charities, Mr. Macilwain never has failed to connect such diseases with constitutional cachexy; and appeals to the experience of his brethren for corroboration of his remarks, that there are innumerable states of the system very remote from health, which yet present "no one of the signs by which we usually recognize disorders." Moreover, that cases are constantly occurring, where consequences excited by local causes admit of no explanation from "the extent or severity of the local injury." It is his firm opinion, that in every case of *spontaneous* local disease, there exists, more or less, derangement of the digestive organs; though he does not think them, "in all cases," *primarily* affected. The *fons et origo mali* often is in the nervous system, with the derangements of which, the digestive organs do not always, but most generally, sympathise. Hence the necessity for merging the treatment of both of these classes of cases into one—the guiding principle being the same—namely, to preserve, or restore, the tranquillity of the nervous system; care being taken, to use Mr. Abernethy's expression, that the nervous system receive no additional disturbance from those organs which are so wont to disturb it, viz. "the chylopoietic viscera." We cannot follow our author into some lengthy digressory remarks, for which however he apologizes; but take him up where he seeks to undeceive those who may have too hastily, or inconsiderately adopted Bateman's opinion of the occasionally exclusive local nature of porrigo.

Though children apparently quite healthy receive it by contagion, that admission does not prove its local character; nor is there any necessity to flee to other reasonings or analogy for its explanation, "in the vast majority of cases" the constitutional disturbance not being so masked as to require it. Even where there was less notable constitutional disturbance, and where the local irritation consequent on extreme neglect seemed sufficient to keep up disease—in vain, his dispensary experience told him, was purely local treatment had recourse to. The indications of disturbed health appear to have been very various;

"But furred, vascular, or otherwise unhealthy tongue; costive, or purged bowels; deficient or voracious appetite; fitful or fidgety state of the nervous system, rendering the patients fractious or unmanageable; slimy, gelatinous, discoloured or otherwise disordered secretions from the bowels; one or more of these several conditions in various combination has been present. Many children have been presented in that peculiar state of unhealthy repletion consequent on protracted lactation, so prolific a source of disease amongst the children of the poor in this metropolis. Some patients have had the abdomen *tumid,* and other marks of mesenteric affection. In fact I have seen but very few who have not presented some indication of disordered health which was far from equivocal. And with regard to those few in whom these indications have been less marked, I can only say that if a child got rapidly well of a troublesome and loathsome disease in less than one-fourth of the time usually required for its removal by a treatment directed to tranquillize the functions of the digestive organs, I should be just as satisfied that it depended on a disturbed condition of these organs, as if the tongue had been ever so furred, or there had been present all the usual symptoms of alimentary or chylopoietic disorder." 23.

Of the reciprocity of sympathy subsisting between the skin and alimentary mucous membrane, many illustrations are hinted at. Mr. Macilwain thinks counter-irritation of the digestive canal is seldom required, "and still more rarely advisable," in conducting the management of diseases of the skin. We have a statement submitted of the success, and the period of time required, attending the dietetic treatment recommended by Mr. Macilwain, from his experience in the Finsbury Dispensary. We only cursorily notice this here, as we mean, towards the close of this article, to contrast the success and duration of time patients were under treatment, with Alibert's experience, in the Hospital of St. Louis. The contrast is striking, and bespeaks strongly the superiority of Mr. Macilwain's plan.

We have next to consider the author's chapter of opinions, as to the prime importance of diet. We think that amplification beyond what was necessary, or even to be expected, has here been indulged in by the author. The whole chapter may be regarded as in a great measure a special commentary on the doctrines of Abernethy.

The powers of the stomach must be ascertained. The food must be proportioned as to quantity, to suit the powers of the stomach. Further, lest there should be imperfect assimilation of the food allowed, choice should be made of that which shall prove least hurtful, in the event of being imperfectly assimilated. The state of appetite is confessedly but an uncertain criterion by which to judge, in all cases, what quantity of food should be allowed. Yet the degree of appetite Mr. M. thinks may be depended on "more generally than is supposed, *provided* that we give the nerves of the stomach a fair chance of exercising their healthy functions, and do not subject them to the misleading influence of unnecessary stimulation."—(p. 30.) Inordinate appetite may more frequently be the result of direct stimulation than disease. Mr. Macilwain illustrates this position by alluding to variety of dishes at table, provoking false appetite by mere change of flavour. "Acid, saccharine, and saline substances are well known to be powerful exerters of appetite," says our author; but we may be pardoned expressing our hesitation to accede to this, without some limitation. We cannot admit "saccharine substances" to be among the number of accredited *whetters* of the appetite. By pleasing the *palate*, we grant a multitude of dainties, such as sweetmeats and confectionary, are transferred from the dessert table to the stomach; yet we cannot allow that such habits of gorging as are practised among the children of the opulent, much more than among those of the indigent classes, are to be ascribed to the fresh cravings of *appetite*. How frequently do we not find the oversight, if we may not call it error, committed, of mistaking and confounding caprices, referable to the palate only, for defective or otherwise morbidly-increased appetite! Many adults of both sexes not only can, but *will*, eat their meals regularly, whether they feel appetized or not. On more occasions than one have we had this sufficient explanation given by such gourmandish individuals, that they ate merely because they supposed "the tabernacle" required being supported: and though this soup and that pastry might perhaps offend their stomach, nevertheless the *palate* relished the variety! But to keep close to our author:—

To avoid repletion from variety of dishes, the use of more than a single article of food is interdicted, during the course of any one repast. Everything stimulant being withheld, using the epithet in its widest dietetic sense. Circumstances, not rules, must determine the quantity of aliment taken at one meal; and during twenty-four hours. If these rules be borne in mind, errors from overhasty generalization will be avoided.

"No man can entertain a more profound respect than myself, for the opinions of Mr. Abernethy, nor more sincerely feel that he has been very largely a benefactor of mankind. Yet I humbly conceive, that the application of his principles, as taught by himself, is not wholly free from the effects of too hasty a generalization. His general rules, I believe to be the very best with which we are acquainted; but I scarcely think that they make sufficient allowance for the exceptions which are met with in practice; and that therefore they are calculated (when too literally or indiscriminately followed) to limit the operation, and consequently to abridge the utility of his own principles." 34.

If repletion be improper, to permit prolonged hunger is no less material an error. Mr. Macilwain's opinions and their applications to practice, though not new, are extremely judicious on this highly important topic.

"Persons who are unnecessarily restricted, become, under the teazing influence of continued appetite, fidgety and uncomfortable. They are impatient and discontented, and influenced by a state of mind exceedingly unfavourable to a healthy condition of body. I must here not be misunderstood: many persons when first subjected to restrictions in diet, will complain of hunger when they have eaten the portion allotted to them, and in half an hour the sensation shall cease. This is not the case to which I refer; as this is morbid sensation, the result of bad habit. It is the continued hunger consequent on an insufficient allowance, which I allude to, as productive of injurious impressions on the nervous system. There are cases truly, in which this plan is necessary, but they are foreign to the present subject. It should be remembered, therefore, that whilst above all things it is necessary to avoid putting more food into the stomach than it can digest, yet it is by no means unimportant to give the organ as much as its powers unassisted by stimulation are fully equal to perform. Now no method with which I am acquainted, is better for this purpose, than first determining how much food we consider necessary for the day's consumption." 36.

Small portions of aliment are recommended at intervals of two, three, four, or six hours, according to circumstances. The periods may cautiously be lengthened; at the same time increasing the quantity till the meals are taken with ordinary frequency, say three times a day, in adults; four times a day for children. Unassimilated food may often be detected by examining the fæces, when otherwise we might search in vain for decisive evidence of indigestion. Imperfect assimilation in other cases may be evidenced by a loaded condition of urine. As we mentioned already, that kind of food should be selected, which, if undigested, shall prove least injurious. Our author prefers vegetable aliment; but we do not altogether clearly see the cogency of his reasons assigned for such preference; viz. that as vegetable aliment ordinarily requires a longer time than animal matters, for the first stages of decomposition, whilst its known tendency to excite the bowels would lead to its more speedy discharge, a consideration of these respective properties should lead us to direct animal food to be avoided, where we have reason to dread the occurrence of chemical changes to which food is exposed, when the organs of digestion and assimilation are in a low state of vitality. Now we do think, if such reasons hold good as applicable to the treatment of porrigo, which we here do not presume to call in question, yet, that granting as much, the case of porrigo is the exception, and not the rule. In no disease ought we to forget that the existing condition of the stomach and viscera, *for the time being*, influences the more or less healthy changes consequent on food being received into the alimentary canal.

To continue the present analysis: Mr. Macilwain then thinks the presence of undigested *farinaceous* aliment occasions less disturbance, and is preferable therefore to animal food. He hazards some observations from which it would seem that he is extremely sceptical; or even broadly denies, that the food undergoes that change in the stomach which the most orthodox of all physiologists have hitherto recognized by the word "chymification!" To avoid error, or unintentional injustice, on our part, towards Mr. Macilwain, we quote, ad longum, his *ipsissima verba.*

"It has been said that the first change which takes place in the food is its conversion into a homogeneous mass, which has been called chyme; but I much doubt whether any such process takes place, and believe this impression must originally have arisen from the observance of farinaceous food, which, when comminuted, and thus exposed to warmth and moisture, certainly presents very much the appearance represented to characterize chyme: no investigation which has been instituted has enabled me to discover any such process. I have opened a great many animals from time to time which had taken food at different periods before death; and many of which had been fed expressly for purposes of investigation after death, but in none did I ever see any thing like the chyme, as it is called. In those fed on purpose, the appearance presented by the meat was, that the surface next the stomach was in a soft gelatinous condition, just such as we might imagine would precede its complete solution, but the remaining portion was just as plainly beef or mutton as it would have been under any other circumstances, making due allowance for its being somewhat obscured by comminition, and the peculiar sort of mawkishly-acid odour of the secretions of the organ. So far as my recollection serves me, the researches of Dr. W. Philip concur in these results. It does not follow that the mode of digestion should be precisely the same in the human stomach: but it is in the highest degree probable that when we find no great difference in structure, there will be a great similarity in function—and therefore that in all animals with membranous stomachs, the process of digestion is essentially the same. It seldom happens that we have an opportunity of examining the human stomach under circumstances which warrant any decided conclusion, but I could never see in the examination of bodies (and I have probably had a fair share of experience in this way), the contents of the stomach representing such an appearance as that usually described under the term chyme. There seems then every reason for concluding that food when undigested in the stomach, retains for a time at least its characteristic properties, and that the idea of its becoming amenable to chemical agency is therefore at least probable." 41.

We beg to leave our readers to draw their own conclusions.

Undue supply of animal food is proverbially a fruitful source of bad health during childhood; a diet, therefore, which is improper in health, cannot but be injurious during disease. In all classes of cutaneous diseases to which childhood is liable, our author judges vegetable food to be that which, cæteris paribus, should form the greater proportion of the diet; and when porrigo is the form of affection, no diet is more suitable or more required than the *farinaceous.* Nevertheless, Mr. Macilwain does not restrict the diet absolutely to what is strictly considered farinaceous—milk, yolks of eggs, bread of different kinds, sago, arrow-root, tapioca, and even "plain puddings," may be conjoined. From such a list as that, we should regard the parent or child indeed difficult to please if the bill of allowed fare were complained of, as not sufficiently ample to give scope to taste or fancy being gratified in the way of selection!

The habit, among some of the better classes, of having their children brought in after dinner to partake of the dessert, is undoubtedly of mischievous tendency. Mr. Macilwain has not exaggerated the bad consequences of such very mistaken kindness. More especially is this practice reprehensible,

"When children have been deprived of this indulgence for a short time, during the more troublesome period of some trifling indisposition, as the milder cases of Porrigo are considered. I need hardly add, that on these occasions, whatever is put into the stomach is to be regarded as trash presented to the organ, when it neither requires nor is prepared for food. All this may perhaps be very natural, and that for which in perfect health good-nature may find excuses; but whilst there is any disorder, surely nothing can be more mischievous, or when forbidden, more inexcusable." 45.

Our author is most particular in urging the strictest observance of his directions as to diet. If these cannot be complied with or are disregarded, "I would never," says he, "continue my advice; for the profession is too often brought into discredit by holding nominally the charge of a case of which it really has only the responsibility."

The potatoe diet of the poor agrees well with children, provided these esculent roots be well-boiled, and be *mealy*.

We see matter for congratulation throughout the profession, that cases of porrigo among the poor are not any of those diseases in which the diet that is necessary cannot be adhered to, if made use of at all, owing to the pecuniary or other circumstances of the sufferers. It would appear that further suggestion as to diet is unnecessary, saving only that the poor should be cautioned against dangers from repletion. We think, if so much as Mr. Macilwain says of his success depended on the scrupulous adherence to farinaceous diet, modified of course, according to the exigencies of individual cases, that practitioners who adopt his suggestions will find few cases intractable in their hands. Is there no hygienic influence to be attributed to the farinaceous diet adopted, not from choice but stern necessity, by our Irish and other poor? As far as the reviewer's experience goes, perhaps severer cases of porrigo, characterized too by greater obstinacy, are oftener met with among a given number of the better classes of society than among a proportionate number of the working and industrious poor (?) Our object however being to keep as closely as possible to the contents of the treatise under notice, we must be sparing of comment, as this article threatens very far to exceed the limits which many circumstances remind us of the expediency to avoid. So much for food. As to the drink recommended, we have —toast and water, barley and water, or plain water.

Plans however excellent in the abstract, often require being introduced only by degrees; and it is not always correct or safe to treat disordered stomachs in accordance with methods suggested by considerations drawn from the same organ when healthy. Thirst should be satisfied as in the case of hunger, that is, before it become urgent; but its first requests should be disregarded. Some of Mr. M.'s patients have been the children of medical men; their cases evinced frequently the usual obstinacy of the disease, although to all appearance under a judicious course of medicine: but "when subjected to a mild farinaceous diet" yielded readily like others. Although diet be the chief part, yet it is a part only of the treatment; disordered secretions must be corrected; sluggish, irritable bowels, excited or regulated.

The consideration of these points brings us to Chapter IV. which is devoted to the *medical* treatment.

If too much food be bad, too much medicine is worse. Medicine taken into the system cannot be followed with negative results : harm must be done if none of the intended benefit be achieved for which drugs are administered. The treatment in all cases should be " commenced by a gentle but efficient evacuation of the bowels."—(p. 54.) Reports of nurses and others should not be trusted to. Small doses of aperient medicine should be given every three or four hours on the Abernethian plan, by which the fæcal contents of the canal will more certainly and effectively be dislodged and discharged, than from the exhibition of large doses which give rise to watery secretions "constituting in many cases an injurious depletion." Almost all medical men are, we think, sufficiently aware of the superior value of reiterated small doses of aperients to full doses of drastic cathartics, in cases where there has been a teazing occasional diarrhœa, and unsatisfactory stools responding to larger doses of medicine. Cases of protracted dyspepsia are sufficiently common; and every one of our standard authors on the medical management of these diseases have bestowed such pains in detailing the best mode of treatment that we cannot but think it unnecessary for any author now-a-days to do more than refer to such works; more particularly as they have a place allotted them in every medical man's library. The cases therefore detailed from pp. 55 to 59, of the man who died after his limb was amputated, and in whose intestines "scybalæ as if of a calculous nature" were found impacted, and the amply detailed case of the ophthalmiæ'd patient, present no interest beyond what their exemplification affords of a principle in the management of constipation, now an exhausted subject, in this point of view.

For gradually and gently soliciting the action of the bowels in the cutaneous diseases of children, our author gives calomel, half a grain; rhubarb, five grains; and of ginger, three, at proper intervals. A solution of manna in warm water, with a little aromatic tincture, has also proved serviceable. Castor oil must not be omitted from the list of useful medicines. Aperients may require being varied. Should rhubarb fail, jalap may be substituted. Compound scammony powder, even aloes and soap act in some cases (we cannot tell why) more efficiently and comfortably than milder means employed.

It is of importance to ascertain what organ is at fault, directing attention to its due regulation. Should it prove obstinate it must be attacked through the medium of other organs with which it sympathises. What are called 'alteratives,' are generally chosen to correct the secretions, when depraved. A repetition of the rhubarb, calomel, and ginger every second or third night is generally sufficient, if the diet be strictly attended to. "Some few patients do very well without any medicine at all." Cases often occurred wherein the stomach had to be interfered with *medically*. The subsidence of voracious appetite has frequently been expedited by small doses of ipecacuanha and antimony; the hippo rarely causing sickness, if the bowels have been frequently evacuated. The secretions of the liver and ducts, upon the whole, seemed to be best managed by hydrarg. cum cretâ. When the dejections indicate paucity of bile, if considerable secretions can be procured, benefit will result, for which purpose calomel and jalap, or calomel with aloes, every three or four hours, are recommended. Rhubarb seems sometimes to occasion pain by the slowness of its operation; the addition of a grain of ipecacuanha, or two or three grains of jalap will generally secure its efficient and comfortable action. If slime, mucus, or jelly pass with the stools, there is nothing better than warm water enemata; three quarters of a pint may be injected every night, or night and morning. In strumous constitutions, frictions of the abdomen: in some mesenteric affections Mr. Macilwain speaks of success from the pustular eruption ("artificial porrigo as I have sometimes termed it") consequent on antimonial inunction. In adults the state of biliary secretion may be such as to require small doses of mercury. Yet idiosyncrasy may contraindicate its employment. Good fresh extract of colocynth with dilute nitric acid, forms the best substitutes in the experience of our author. For the detail of much that may be by some regarded as elementary truisms, the usual obstinacy of porrigo, and the success of our author's practice are urged in mitigation of criticism.

From the summary which we have given of the contents of the first four chapters, the reader will have correctly anticipated, that but few pages (11) are devoted by Mr. Macilwain to the *local treatment* of porrigo; with an analysis of the last chapter we shall close this already too extended notice.

Local treatment is not "a thing of no consequence," though little importance is to be attached to the all but innumerable list of topical remedies. Irritation, mechanical or chemical, must be avoided. Whatever has a tendency to confine the discharge must be obviated. The local treatment should be conducted thus:—Before the razor can be used, a large bread and water poultice (without lard or greasy matter) should be applied to the head. Another should follow before the first shall have dried. In this way, incrustations will be removed. Plucking the hair where it is matted together may be, and often is, preferable to shaving such spots, owing to the tenderness of surface. Head must be closely shaved; cases no doubt exist, where this operation of the barber gives rise to much irritation, yet the irritation consequent on shaving less retards recovery than the greater irritation consequent on the growth of hair, if allowed to shoot up beyond the cranial surface. Shaving should be cautiously conducted. The scalp should next be washed with soap and water, till every particle of discharge be removed; then clean tepid water, if liberally used, will remove any soap, after which the surface should be "patted (not rubbed) with soft linen until perfectly dry" (p. 80.) The head should be shaved, say twice a week. *Any* unctuous, slightly stimulating ointment, previously softened by warmth, may be lightly "painted" on the surface by means of a suitable camel-hair brush. The dressings should be repeated once every twenty-four hours, should the discharge not be profuse—night or morning in the latter case, carefully cleansing the surface of the ointment previously employed. Spermaceti ointment is a good general application; but unguentum hydrarg. nitratis, variously diluted, is preferable. Mr. Macilwain begins with a drachm, diluted with an ounce of lard, the strength to be gradually increased. Disposition to local amendment will, however, generally be seen to be in accordance with improvement of the general health. Of sedative, stimulant, or astringent lotions our author has little experience. In conclusion, he observes that—

"The local treatment is simple, yet that each part of it should be carefully executed, regardless of the trouble it requires. The head should be covered with a clean linen cap, and the usual caution of separate beds, towels, basins, &c. observed, to prevent the communication of the disease to other children.

Exercise and pure air, as tending to improve the general health, are no doubt good additions to the plan which I have recommended;—but the obstacles in the way of forming any opinion on the effect of advice directed to the attainment of these objects in a crowded metropolis, put it out of my power to speak from experience with regard to them." 83.

We have perused Mr. Macilwain's treatise most carefully, and have given a fair analysis of its contents. After glancing at the title-page, &c. we were led to form no expectations which have not been realized. The purpose of this little work being avowedly practical, we were not disappointed at finding the author silent as to his experience (if any) of the truth of the occasional influence exerted by some forms of porrigo in retarding, or otherwise modifying, the development of the sexual organs. Cases mentioned by M. Alibert are curious and interesting to the physiologist, but we must content ourselves with merely alluding to them here. Mr. Macilwain is also silent as to the periods of life between which the greatest number of children are afflicted with any of the many forms of porrigo. The assigned interval, from the 2d to the 7th year, we believe, on the whole, to be correct—assuredly it is so far as tinea is concerned. It is gratifying to learn from Mr. Macilwain, that the ordinary duration, under his modified and improved mode of treatment, was so limited and steady, " not more than six weeks" being the average—and some were cured much within that period. Other cases, more obstinate, such as porrigo *decalvans*, " if, indeed, it is to be regarded as porrigo at all" (p. 25), took a longer time for its cure. The superiority of the author's plan must appear conspicuous, when we contrast the regularity and permanence of his success with the results of Alibert's vast experience in the management of the same diseases. Mr. Macilwain, we think, has not done himself justice, in refraining from submitting a table of comparisons, odious though *abuse* in this way proverbially is. We shall, therefore, do it for him by simple statement.

When remarking upon the want of success attendant on the use of *any external applications*, particularly from that savage custom of treating tinea, by clapping a pitch plaster upon the head, and, after securing its adhesion, tearing it off *vi et armis*, accompanied with layers of incrusted matter, and even denuding the bones of the cranium:—from this Mohawk-*scalping* system, and other local treatment, Alibert admits that some were three years!!! a great many twelve months!! and all commonly six months under treatment. After all, relief was any thing but permanent, *rechutes* and *recidives*, (relapses and recurrence), and " more serious maladies," frequently ensued. Need we wonder at the French monographists, under the influence of such obvious suggestions, as failure inculcated, seeking to impress upon their readers the importance of attention to the general health—regulation of the secretions—and a sparing application of local means? No local treatment can be uniformly applicable, nor can any routine system be rational, for the obvious reason, that every form of skin disease has, more or less, an inherent tendency to run through its stages of manifestation, acmé, and decadence.

Before dropping the pen, we must beg Mr. Macilwain, if a second edition afford the opportunity we trust awaits him, to recast the expression of some sentences in his book, which, in their present state of *syntax*, have occasioned us to rub our eyes and reconstrue them more than once, without a satisfactory comprehension of their meaning rewarding us for our pains. In the table of contents, we find this somewhat glaring inconsistency—"the history of cutaneous affections shews them, for the most part, to be *tractable* in nearly the same proportion as they are unconstitutionally treated" (! ?) Indeed! are we to understand that skin diseases, if *im*properly treated, are proportionally *curable*? Again, at page 19—"when I use the term general *health* and *derangement* of the *chylopoietic* viscera, I would not be understood as *always* using them *synonimously*." Really we hope not; and, begging our author's pardon, we think no student runs much risk, if he be in his senses, of misunderstanding *health* and *disease* so far as to regard them as *synonimous* or convertible terms. These trifling and self-rectifying imperfections must, however, be overlooked, and merged in the merits of the publication as a whole, though a more finished style was certainly to have been looked for, from an author who has been already oftener than once before the public. We should consider ourselves as wanting in candour did we fail to recommend this little treatise; we can conscientiously applaud its purpose, and vouch for its useful tendency.

